# Outcomes in clinical trials on sarcopenia: a systematic review and meta-analysis

**DOI:** 10.1016/j.jnha.2026.100821

**Published:** 2026-03-03

**Authors:** Michaela Rippl, Katharina Müller, Sebastian Martini, Ralf Schmidmaier, Olivia Tausendfreund, Sabine Schluessel, Linda Deißler, Michael Drey

**Affiliations:** Department of Medicine IV/Geriatrics, LMU University Hospital, LMU Munich, Ziemssenstr. 5, 80336 Munich, Germany

**Keywords:** Geriatrics, Sarcopenia, Common endpoints, Patient reported outcomes, Clinical trials

## Abstract

**Background:**

In order to improve the quality and comparability of intervention studies, common endpoints have gained significance. The European Society for Clinical and Economic Aspects of Osteoporosis, Osteoarthritis and Musculoskeletal Diseases (ESCEO) consensus paper identified suitable endpoints for trials investigating the treatment of sarcopenia.

With the help of a systematic review, we analyzed which endpoints have been used in randomized controlled intervention trials investigating the treatment of sarcopenia to date, to what extent these correspond to the recommended endpoints of the ESCEO consensus and whether minimal clinically important differences (MCID) were defined. Based on these identified endpoints we also aimed to identify which type of intervention may be most effective in enhancing the investigated endpoint.

**Methods:**

A systematic search (PROSPERO ID: CRD42023468128) was carried out in various databases (PubMed, Embase, PsychInfo, Web of Science, Trial Registers) to find studies meeting the following criteria for further analysis: randomized controlled trials (RCTs), geriatric study population, sarcopenia (including probable sarcopenia and sarcopenic obesity), intervention study, published until October 2023 and written in English. The meta-analysis was conducted for the most common outcomes.

**Results:**

Ninety publications met the criteria and were analyzed as full texts. The 90 intervention studies used a variety of different diagnostic criteria, intervention types and study settings. Measures of physical performance/strength (85 trials) and body composition (77 trials) were the most commonly used outcomes; in 74 studies, both outcome categories were used. Quality of Life was used in only 13 studies. In all categories, a very high heterogeneity between the measures was found and MCIDs were only rarely defined.

Meta-analysis showed significant moderate intervention effects for the Timed-Up and Go-Test (TUG) and appendicular skeletal muscle index (ASMI), Handgrip strength and Chair Rise Test (CRT). The Short Physical Performance Battery (SPPB) showed no significant effects.

**Conclusion:**

Our results highlight the heterogeneity among sarcopenia intervention studies, which impairs the comparability regarding the efficiency of different interventions. However, our findings support the need for core outcomes or pre-defined common endpoints, for which the updated ESCEO recommendations can serve as a solid basis. Nevertheless, more consistent data are needed to enable a proper meta-analysis.

## 1 Introduction

Sarcopenia describes an age-related loss of muscle mass and especially muscle function [1]. Depending on the applied definition approximately 10–27% of the world-wide population are affected [2]. For patients untreated sarcopenia can lead to numerous consequences such as falls and fractures [3], impairments in abilities of daily living [4], loss of independence [5] and it is also associated with multiple comorbidities [6,7]. Also, for society, sarcopenia poses a huge socioeconomic burden as for example hospital costs have been shown to be several times higher in sarcopenic than in non-sarcopenic patients [[8], [9], [10]].

In the course of time also sub-groups of sarcopenia such as probable sarcopenia [1] or sarcopenic obesity [11] were defined. These conditions describe special characteristics of sarcopenia as for example in patients with sarcopenic obesity the overall muscle mass might be elevated but is still too low with regard to their increased body fat [11]. In patients with probable sarcopenia only a limitation in muscle function but no reduction of the muscle mass can be detected [1]. As a reduced muscle function is the driving characteristic of sarcopenia, probable sarcopenia is treated equally to the overall picture of sarcopenia [1].

Therapeutic options for all conditions mainly consist of nutritional and physical training interventions [1] since despite ongoing medical trials no medical treatment has yet prevailed [12]. In probable sarcopenia and sarcopenia physical training has been shown to be most beneficial compared to nutritional interventions alone [13] whereas especially in patients with sarcopenic obesity a combination of physical training and nutritional interventions seems advantageous [14]. However, for the evaluation and comparability between different interventions common outcomes are needed [15] but until now no core outcome sets have been defined, leading to the use of many heterogenous endpoints [16]. In 2021 the European Society for Clinical and Economic Aspects of Osteoporosis and Osteoarthritis (ESCEO) published updated recommendations for clinical trials in sarcopenic patients in which adequate endpoints and measurement tools were defined [15].

The minimal clinical important difference (MCID) offers a quantifiable method for measuring the effects of an intervention on the desired endpoint [17] but especially in geriatric patients suitable MCID reference values are still lacking [18]. Multimorbidity, the wide heterogeneity among geriatric patients regarding their preserved abilities and health status, and a limited life expectancy are possible reasons for this [18].

The goal of this systematic review was to identify which endpoints have been used in randomized controlled trials (RCTs) on the treatment of sarcopenia, whether MCIDs were defined and whether the chosen endpoints were in line with the ESCEO recommendations.

## 2 Methods

This systematic review was conducted according to the Preferred Reporting Items for Systematic Reviews and Meta-Analyses (PRISMA) criteria [19]. This review has PROSPERO registration, with the complete research protocol accessible for consultation (ID: CRD42023468128).

### 2.1 Search strategy and study selection

Literature search was conducted in October 2023 in MEDLINE (via PUBMED), EMBASE (via Ovid), Web of Science, PsychInfo and clinical trials registers (ICTRP, ClinicalTrials.gov., CINAHL). The search strategy including search terms can be found in the supplementary material (Table S2). No publication date limitation was applied; the search covered all available records up to and including October 2023.

Studies were included if they met the following criteria:

1 Older adults (aged 60/65 years and older, depending on the applied sarcopenia definition) with diagnosed probable sarcopenia, sarcopenia or sarcopenic obesity according to EWGSOP, EWGSOP2, AWGS, FNIH or similar
2 Interventional trial: all kinds of interventions with the aim to treat sarcopenia, including drugs, nutrition, exercise / physical training
3 Sarcopenia as the main condition for the intervention
4 Randomized controlled trial: comparators without treatment, standard care or placebo
5 Published in English.

Both reviewers performed the literature search according to the predefined criteria separately. Any disagreements were resolved through discussion. Duplicates were excluded by Mendeley reference manager tool. The study screening process was carried out in two stages: Initially through title and abstract review and subsequently through full-text evaluation. Both reviewers applied the eligibility criteria and selected studies for inclusion in the systematic review. Systematic Review Data Repository-Plus (SRDR+; accessed at https://srdr.ahrq.gov/) was the tool used for the selection process, data extraction and risk of bias assessment to ensure a standardized, transparent, and collaborative process. The platform is designed for systematic reviews and compliant with PRISMA, and facilitates quality assurance and traceability through integrated protocol and export functions.

### 2.2 Data extraction and analysis

Data on study design, participants’ demographics, sarcopenia diagnostic criteria and outcome variables including information on MCIDs were extracted from the study documents.

To assess the methodological quality and risk of bias of the eligible studies we conducted additional analysis using the Cochrane risk of bias tool (RoB) [20], as implemented in SRDR+. This framework facilitates a structured evaluation of potential sources of bias that may arise at different stages of a trial.

Meta-analyses were conducted for selected outcome variables, focusing on the most frequently reported measures of body composition (appendicular skeletal muscle index, ASMI), muscle strength (Handgrip strength and CRT) and muscle function (short physical performance battery, SPPB and Timed-Up and Go–Test, TUG). To ensure comparability across studies, only studies reporting both, pre- and post-intervention values as means and standard deviations (mean/SD) for intervention and control groups were included in the meta-analyses. Prior to conducting the meta-analysis and meta-regression using SPSS (IBM SPSS v.29.0, IBM Corp, Armonk, NY, USA), the effects (Cohens d) were calculated. In studies including more than two intervention groups, each intervention group was compared separately with the control group, rather than comparing intervention groups with one another. To address the potential issue of double counting when multiple intervention groups were compared with a single control group, the sample size of the control group was divided proportionally across the respective intervention comparisons. This approach maintains the statistical independence of effect size estimates while allowing all relevant intervention arms to be retained in the analysis. The means and standard deviations of the control group were left unchanged, and only the sample size was adjusted accordingly. This procedure is explicitly recommended in the Cochrane Handbook for Systematic Reviews of Interventions [21]. If multiple post-intervention assessments were reported, the result closest to the end of the intervention was used.

Effect sizes (Cohen’s d) were calculated using the formula proposed by Morris [22] and implemented via the online tool by Lenhard & Lenhard [23]. This method was selected because several included studies exhibited relevant baseline differences between intervention and control groups, which needed to be accounted for in the effect size estimation. Accordingly, effect sizes (d) were computed as the standardized between-group difference in change scores, thereby adjusting for pre-existing baseline differences. Standardization was based on the pooled pre-test standard deviation. This approach was applied under the assumptions that (i) pre–post change scores provide a less biased estimate of the intervention effect in the presence of baseline imbalances, (ii) measurement scales and reliability are comparable across time points, and (iii) standardization using the pooled pre-test standard deviation yields a conservative and stable effect size estimate. Under these assumptions, post-test–only standardized mean differences were considered less appropriate, as they do not explicitly adjust for pre-intervention group differences and rely on stronger assumptions regarding baseline equivalence. In addition, the standard errors [24] were calculated.

With regard to methodology, it should be noted that in some cases our calculated effect sizes differed from those reported in the original studies. These discrepancies may be attributable to differences in analytical strategies, including the fact that our analyses focused on pairwise comparisons between intervention and control groups, whereas several original studies reported omnibus group tests (e.g., ANOVAs). Furthermore, by applying the Morris method, we adjusted for baseline differences, which may not have been accounted for in the original analyses. Additional contributing factors may include differences in study weighting and the wider confidence intervals that result from pooling multiple studies in the meta-analysis.

A random-effects model was applied to estimate the pooled effect size, accounting for both within-study and between-study variance. Between-study variance (τ²) was estimated using the restricted maximum likelihood (REML) method. Statistical heterogeneity was assessed using the Q statistic, and the I² index, with I² values of 25%, 50%, and 75% interpreted as low, moderate, and high heterogeneity, respectively. Publication bias was assessed using Egger’s regression test and the trim-and-fill method under a random-effects framework. To explore potential sources of heterogeneity, random-effects meta-regression analyses was conducted when at least 10 studies were available [25]. Moderator variables included intervention type, diagnostic criteria for sarcopenia, sample size, and publication year. The regression models employed the Knapp–Hartung adjustment to obtain robust standard error estimates. Statistical significance set at p < 0.05. Results are reported as pooled effect sizes with corresponding 95% confidence intervals, along with heterogeneity measures.

## 3 Results

As 90 studies were analysed in this systematic review, for clarity reasons no citations were added in the following. Results with references are shown in the Supplement Table S1.

### 3.1 PRISMA flow-chart – study selection and characteristics

**Fig. 1:**
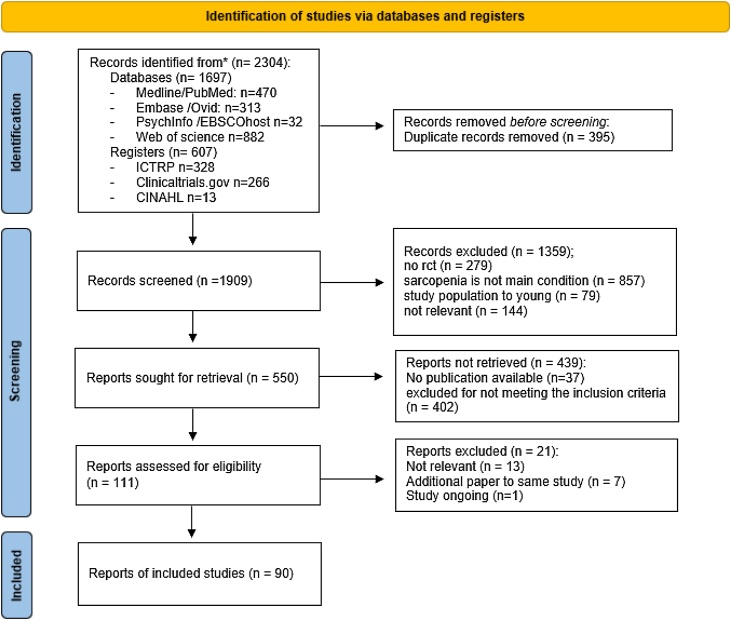
PRISMA Flow chart.

A total of 2304 potentially eligible articles were identified from databases (470 articles from Medline/PubMed, 313 articles from Embase/Ovid, 32 articles from PsychInfo/EBSCOhost, 882 article from Web of science) or registries (328 articles from ICTRP (International Clinical Trails Registry Platform), 266 articles from Clinicaltrials.gov and 13 articles from CINAHL) using our search strategy. The automated duplicate search included in Mendeley identified 395 duplicate articles which we therefore removed. Title screening was conducted in the remaining 1909 articles, leading to the exclusion of 1359 articles as either the study design did not meet the criteria for a randomized controlled trial (279 articles), or sarcopenia was not the main condition for treatment (857 articles), or the study population was too young (79 articles) or for other reasons (144 articles). 550 articles were found to be suitable for abstract screening of which 439 were excluded as 402 articles did not meet the inclusion criteria and for 37 articles no full-text publication was available. We performed full-text screening in the remaining 111 articles, leading to the inclusion of 90 studies, comprising 9117 patients. The literature review process is shown in Fig. 1 Flow chart.

#### 3.1.1 Risk of bias assessment

To assess the risk of bias of the included studies, the Cochrane Risk-of-bias tool included in SRDR+ was used.

**Fig. 2:**
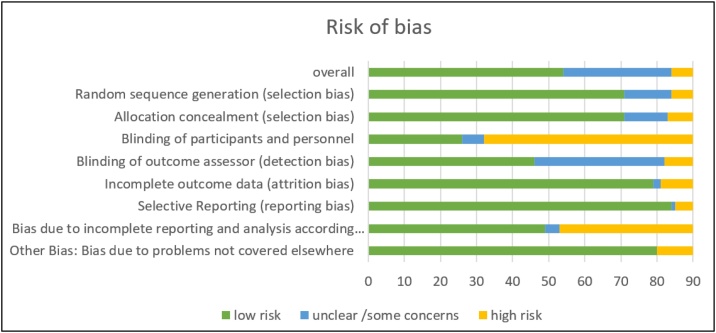
Risk of bias assessment. Displayed is a plot illustrating the number of studies with low, intermediate and high risk of bias in each assessment domain identified by the reviewers.

The risk of bias assessment revealed notable variations across different domains (see Fig. 2 Risk of bias). Overall, 54 studies were classified as having a low risk of bias, 30 studies showed some concerns, and 6 studies were rated as high risk.

Selective reporting bias was the least concerning, with 84 studies rated as low risk and only 5 classified as high risk. Similarly, incomplete outcome data (attrition bias) and allocation concealment showed a predominantly low risk, with 79 and 71 studies rated as low risk, respectively.

However, significant concerns were identified in blinding-related domains. Only 26 studies had a low risk of performance bias (blinding of participants and personnel), while 58 studies were classified as high risk. Detection bias (blinding of outcome assessors) also showed considerable uncertainty, with 36 studies rated as unclear or having some concerns. The low proportion of studies with adequate blinding can largely be attributed to the nature of the interventions examined. Since many of the included studies applied physical training interventions, full blinding of participants and personnel is often not feasible. Participants in exercise interventions are typically aware of their assigned training program, which inherently increases the risk of performance bias.

Intention-to-treat analysis and random sequence generation presented moderate risks, with 49 and 71 studies rated as low risk, but also a notable proportion categorized as high risk (37 and 6, respectively).

### 3.2 Descriptive analysis

#### 3.2.1 Population (see supplement Table S1)

55 studies included community-dwelling patients, 5 studies included hospitalized patients, and 9 studies included patients living institutionalized. In 18 studies patients from other cohorts were included and 3 studies did not declare the type of included patients.

#### 3.2.2 Publication date (see supplement Table S1)

Most of the included studies were published between 2019 and 2023 with a maximum of 14 included studies in 2019 and 2022. The lowest number of included studies was found in the years 2012, 2014 and 2015 by only two studies per year.

#### 3.2.3 Continents (see supplement Table S1)

The majority of the included studies (n = 46) was conducted in countries assigned to the Asian continent. Studies from Europe represented the second most common origin (n = 25). South- (n = 8) and North-America (n = 6) contributed the third most common portion. Studies from Australia (n = 1) and Africa (n = 1) were sparse whereas 3 studies were conducted across multiple continents.

#### 3.2.4 Diagnosis criteria and sarcopenia status (see supplement eFig. S1a)

Asian Working Group for Sarcopenia (AWGs)- (n = 22) [26] and European Working Group on Sarcopenia in Older People (EWGSOP)- (n = 26) [27] criteria were the most commonly used definitions for sarcopenia status. AWGS2 criteria [28] were applied in 6 studies and EWGSOP2 criteria [29] were applied in 4 studies. In 4 studies the Foundation for the National Institutes of Health (FNIH) criteria [30] were applied. 24 studies were based on other, less common sarcopenia definitions such as the definition by Baumgartner et al. [31] or otherwise pre-defined cut-off values for muscle power or function and muscle mass (e.g., residual method by Delmonico [32] or physical performance tests e.g., SPPB 3–9 points [33]). 2 studies used combined EWGSOP2+AWGS-criteria and 2 other studies used combined EWGSOP + AWGS criteria.

#### 3.2.5 Type of included Sarcopenia status (see supplement eFig. S1b)

61 studies included sarcopenic patients only. In 3 studies only patients diagnosed with probable sarcopenia and in 7 studies patients with both, sarcopenia or probable sarcopenia were included. 18 studies included patients with sarcopenic obesity and in 1 study patients with sarcopenia or sarcopenic obesity were included.

#### 3.2.6 Body composition measurement (see supplement Table S1)

Most of the included studies either used DXA (n = 35) or BIA (n = 39) for body composition measurement. Four studies indicated using both methods. Other methods such as Magnetic Resonance Imaging (MRI) or Computer Tomography (CT) were rarely used. 4 studies used other tools such as calf circumference measurement, 5 studies used combined measurement tools (e.g., DXA + BIA) while 1 study did not state the applied method.

#### 3.2.7 Intervention type (see Fig. 3)

Exercise (n = 35) was the most common intervention type followed by a combination of nutritional and exercise interventions in n = 30 studies. Nutrition-only interventions were conducted in 13 studies. Some studies combined exercise and nutrition with other interventions (n = 1) or combined exercise with drug (n = 1) or other interventions (n = 1). Nutrition and other interventions were used in 2 studies. In 2 studies medication was used as the only intervention, one study combined medication with nutrition and another combined medication with nutrition and exercise. 3 studies used other forms of intervention.

**Fig. 3:**
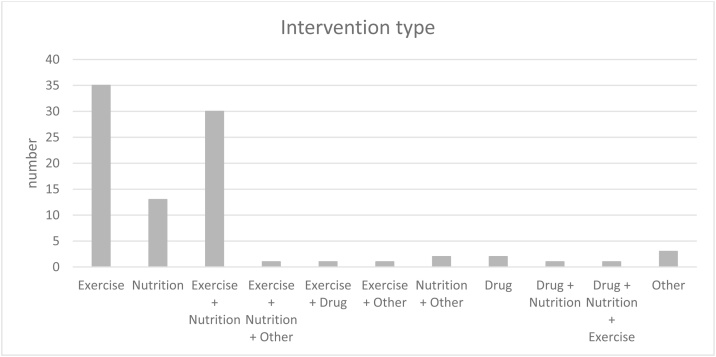
Illustrates the types of interventions used and how many studies applied each type.

#### 3.2.8 Primary and secondary outcomes (see Fig. 4)

The outcomes were categorised into 4 groups: (1) body composition, (2) physical performance and strength, (3) Quality of Life (QoL) and (4) other (e.g., laboratory findings, activities of daily living, cognition).

**Fig. 4:**
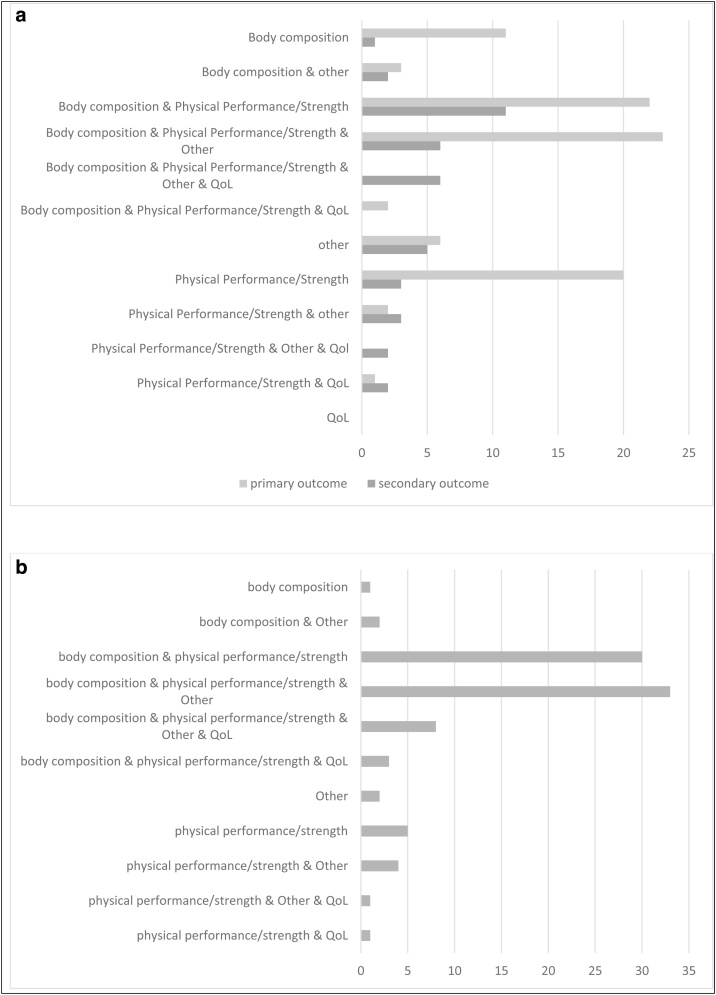
(a) Shows the number of studies using different categories as primary or secondary outcomes. (b) Shows the number of studies combining different outcome categories.

Most studies defined a combination of different measurements as primary outcomes. The most frequently reported primary outcome domains were body composition (n = 61) and/or physical performance/strength (n = 70). QoL measures were designated as primary outcomes in only three studies, and exclusively in combination with other otucomes.

In 34 studies other variables were included, most often in combination with body composition and/or physical performance measures.

Secondary outcomes were defined in 41 studies; and included body composition measures (n = 25), physical performance/strength outcomes (n = 31), QoL tools (n = 11) and other variables (n = 24).

In total in 85 trials (see Fig. 4b), measures of physical performance/strength and in 77 studies body composition parameters were part of the outcome set (either as the primary or secondary outcome); in 74 studies both outcome categories were used. QoL was a primary or secondary outcome in 13 studies only.

#### 3.2.9 Physical performance and strength (see supplement eFig. S2)

The most frequently applied measures for muscle strength and physical performance were handgrip strength (n = 63), gait speed (n = 39), CRT (n = 25), TUG (n = 22), SPPB (n = 22), different walking tests (n = 17), knee extension strength (n = 16) and balance tests (n = 5). 30 trials applied different measures than the ones mentioned previously.

The assessment of these variables was highly heterogeneous across studies. Even for the aforementioned outcomes, substantial variation existed in the applied measurement protocols. For example, the CRT was implemented using different formats, including the "5 Times Sit-to-Stand," the "30-Second Chair Stand," and the "Rising from Sitting Position Test." Similarly, walking tests differed with respect to distance and pace, with studies employing, 4-meter, 6-meter, or 10-meter protocols, assessed either at usual or maximal walking speed.

#### 3.2.10 Body composition (see supplement eFig. S3)

Measurements for body composition were very heterogenous. In total 28 different measures or combinations of these were applied as body composition outcomes. Most frequently used outcomes were Skeletal Muscle Index (SMI; n = 22), Body Mass Index (BMI, n = 21), Fat Free Mass (FFM; n = 14) and ASMI (n = 13). In summary 29 different muscle mass measures were applied and in 69 trials at least one of them was included as an outcome. Seven varieties of fat mass measures were identified and in total 18 studies applied any of these measures.

#### 3.2.11 Quality of life

In 13 studies Quality of life measures were identified as a primary or secondary outcome. The most common measures were the EQ5D (n = 5) and the SF 36 (n = 5). In two studies both were combined. Two studies applied the SF 12. Only two trials included the sarcopenic specific health related Quality of life Questionnaire (SarQoL).

#### 3.2.12 Other

In 50 studies other measures were defined as primary or secondary outcomes. ‘Other’ measures comprised laboratory prameteres (e.g.,IGF-1, myostatin, glycemia, inflammatory markers, anaemia biomarker), nutritional assessments (including MNA), ADL, IADL, falls, mortality, pain, mood, depression, and cognitive function.

#### 3.2.13 MCID

Supplementary table 2 provieds a detailed description of all parameters for which MCID thresholds were applied, as well as the studies the corresponding studies. MCIDs for physical performance measures were reported in 8 studies whereas MCIDs for body composition measures were applied in 6 studies. Only Murphy et al. (2022) used MCIDs for both physical performance and body composition outcomes.

The most frequently applied MCID was for SPPB MCID, reported in 4 studies, followed by gait speed (n = 3) and fat free mass (n = 2). MCIDs for composite strength, TUG, quadriceps strength, handgrip strength, 6-min. walking distance, Barthel Index, Sarcopenia Z-Score and appendicular lean mass MCIDs were applied in only one study.

### 3.3 Meta analysis

The selection of outcomes for the meta-analysis was based on frequency with which measures were used within the three predefined outcome categories: body composition, muscle strength, and muscle function. Within the body composition (BC) domain, the ASMI was the most frequently reported outcome. Muscle strength was primarily assessed using handgrip strength and CRT, whereas muscle function was most commonly evaluated by SPPB and the TUG. Only the studies providing the necessary data were eligible for inclusion in the meta-analysis.

**Fig. 5:**
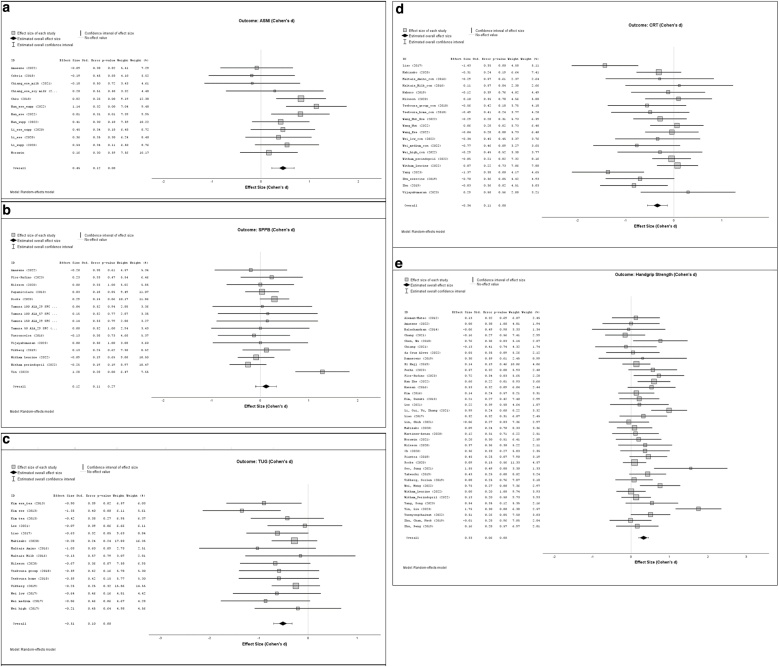
(a) Forest plot showing the results of the meta-analysis on ASMI (appendicular skeletal muscle index). (b) Forest plot showing the results of the meta-analysis on SPPB (short physical performance battery). (c) Forest plot showing the results of the meta-analysis on TUG (Timed Up and Go Test). (d) Forest plot showing the results of the meta-analysis on CRT (Chair Rise Test). (e) Forest plot showing the results of the meta-analysis on Handgrip strength.

Forest plots (Fig. 5a–e) display the effect sizes of the different interventions for the respective outcomes. In each plot, individual study effect sizes are represented by squares, with horizontal lines indicating the corresponding confidence intervals. Diamonds represent the pooled overall effect estimates.

#### 3.3.1 ASMI

A total of 12 groups out of 8 studies were included in the quantitative synthesis. A random-effects model was applied to account for between-study variability. The pooled analysis yielded a significant overall effect size of d = 0.452 (SE = 0.11, 95% CI [0.255, 0.679]), z = 3,895, p < 0.001, indicating a moderate effect across studies.

The results indicated a significant publication bias, as evidenced by Egger’s test (p = .036) and the trim-and-fill analysis, which imputed three missing studies.

The test of homogeneity was not significant, Q(11) = 14,747 p = 0.194, suggesting no notable heterogeneity among the included studies.

A meta-regression to explore potential sources of the heterogeneity was not performed, as fewer than 10 studies were available, rendering such án analysis methodologically inappropriate [25].

#### 3.3.2 SPPB

A total of 15 groups out of 11 studies were included in the quantitative synthesis. A random-effects model was applied to account for between-study variability. The pooled analysis yielded no significant overall effect size of d = 0.118 (SE = 0.1080, 95% CI [-0.093, 0.330]), z = 1.095, p = 0.273, indicating no significant effect across studies. The Egger’s test indicated no significant publication bias (p = .618).

The test of homogeneity was significant, Q(14) = 24.532 p = 0.039, suggesting notable heterogeneity among the included studies. Correspondingly, heterogeneity indices indicated a moderate proportion of true variance (τ^2^ = 0.074; I^2^ = 49,2%).

A random-effects meta-regression was subsequently conducted using the restricted maximum likelihood (REML) estimation method with Knapp–Hartung standard error adjustment to explore potential moderators (intervention type, diagnosis algorithm, publication year, and sample size). The overall model was not statistically significant, F(10, 4) = 3.289, p = 0.131, indicating that the included predictors did not explain a significant portion of the heterogeneity.

#### 3.3.3 TUG

A total of 15 groups out of 10 studies were included in the quantitative synthesis. A random-effects model was applied to account for between-study variability. The pooled analysis yielded a significant overall effect size of d = −0.511 (SE = 0.096, 95% CI [−0.699, −0.324]), z = −5,34, p < 0.001, indicating a moderate effect across studies. The Egger’s test indicated no significant publication bias (p = .983).

The test of homogeneity was not significant, Q(14) = 11,306 p = 0.662, suggesting no notable heterogeneity among the included studies.

#### 3.3.4 CRT

A total of 20 groups out of 12 studies were included in the quantitative synthesis. A random-effects model was applied to account for between-study variability. The pooled analysis yielded a significant overall effect size of d = -0.342 (SE = 0.10, 95% CI [-0.549, -0.135]), z = -3,239, p = 0.001, indicating a moderate positive effect across studies. The Egger’s test indicated no significant publication bias (p = 0.995).

The test of homogeneity was significant, Q(19) = 33,591, p = 0.021, suggesting notable heterogeneity among the included studies. Correspondingly, heterogeneity indices indicated a moderate proportion of true variance (τ^2^ = 0.094; I^2^ = 45,6%).

A random-effects meta-regression was subsequently conducted using the restricted maximum likelihood (REML) estimation method with Knapp–Hartung standard error adjustment to explore potential moderators (intervention type, diagnosis algorithm, publication year, and sample size). The overall model was not statistically significant, F(8, 11) = 1.523, p = 0.254, indicating that the included predictors did not collectively explain a significant portion of the heterogeneity. After adjusting for these moderators, residual heterogeneity decreased to a moderate level (τ^2^ = 0.03; I^2^ = 20.4%), indicating that about half of the between-study variability remained unexplained.

#### 3.3.5 Handgrip strength

A total of 37 groups out of 36 studies were included in the quantitative synthesis. A random-effects model was applied to account for between-study variability. The pooled analysis yielded a significant overall effect size of d = 0.33 (SE = 0.064, 95% CI [0.201, 0.45]), z = 5.128, p < 0.001, indicating a moderate positive effect across studies. The Egger’s test indicated no significant publication bias (p = 0.844).

The test of homogeneity was significant, Q(36) = 66.516 p = 0.001, suggesting notable heterogeneity among the included studies. Correspondingly, heterogeneity indices indicated a moderate proportion of true variance (τ² = 0.64; I² = 46,1%).

A random-effects meta-regression was subsequently conducted using the restricted maximum likelihood (REML) estimation method with Knapp–Hartung standard error adjustment to explore potential moderators (intervention type, diagnosis algorithm, publication year, and sample size). The overall model was not statistically significant, F(11, 25) = 1.059, p = 0.429, indicating that the included predictors did not collectively explain a significant portion of the heterogeneity. Among individual moderators, none of the predictors significantly influenced the effect size. After adjusting for these moderators, residual heterogeneity decreased a little (τ^2^ = 0.061; I^2^ = 43.2%), indicating that most of the between-study variability remained unexplained.

## 4 Discussion

The 90 included intervention studies demonstrated substantial heterogeneity with respect to diagnostic criteria, intervention types and study settings. The most frequently assessed outcomes were physical performance/muscle strength (85 trials) and body composition (77 trials); with both outcome categories reported in 74 studies. In contrast, Quality of Life (QoL) was assessed in only 13 studies.

Despite ESCEO`s recommendations [1], most studies relied on combined endpoints incorporating muscle strength, physical function, and body composition, rather than prioritizing physical performance and patient reported outcomes (PROs) [34].

One explanation may be the widespread availability of well-established objective tools for assessing body composition, which provide quantifiable and reproducible results. In contrast, PRO instruments are still gaining acceptance and may be perceived as inherently subjective [29,35]. Methodological concerns – such as limited sensitivity to change and the lack of consensus on minimal clinically important differences (MCIDs) - further complicate their use and interpretation in intervention trials [36]. Practical considerations, including increased time requirements and the need for trained personnel to ensure standardized administration, may additionally limit PRO implementation, particularly in large or resource-constrained studies.

Among studies that included PROs, SF-36 and EQ-5D predominated, whereas sarcopenia-specific QoL-measures (e.g., SarQoL [37]), remain underutilized. Given that ESCEO's recommendations explicitly endorsing PROs were only introduced in 2021, their limited uptake is not unexpected. Importantly, PROs are intended to complement objective performance-based measures by capturing patient-centred benefits not fully reflected by changes in muscle mass or strength alone. This perspective aligns with the recently published GLIS (Global Leadership Initiative in Sarcopenia) statement, which highlights QoL as a key outcome in sarcopenia research, particularly in observational contexts [34]. Although intervention trials differ in scope, the growing recognition of QoL may encourage broader and more consistent inclusion of PROs in future sarcopenia interventions.

A related challenge affecting comparability and clinical interpretability is the inconsistent application of MCIDs [17]. While MCIDs are available for some physical performance and body composition measures, they remain poorly defined for most PROs [38]. Only 14% of included studies reported MCID use, predominantly for physical performance and muscle mass/body composition outcomes. This limits the ability to determine whether statistically significant effects translate into clinically meaningful benefits, thereby constraining the applicability of the meta-analytic findings for clinical decision-making. Establishing standardized MCIDs for sarcopenia intervention trials – while accounting for variability across populations and settings – represents an important priority for future research [[33], [34], [35], [36]].

Against this background, the present meta-analysis demonstrates differential intervention effects across functional and morphological outcomes, with moderate improvements observed in several domains, albeit accompanied by varying degrees of heterogeneity and limited explanatory power of study-level moderators.

For appendicular skeletal muscle mass index (ASMI), a significant moderate pooled effect (d = 0.45) indicated beneficial structural adaptations. Although between-study heterogeneity was negligible, evidence of publication bias was detected, with trim-and-fill analyses imputing three potentially missing studies. Consequently, the magnitude of the pooled effect may be overestimated and should be interpreted with caution. Meta-regression was not performed due to the limited number of available studies.

In contrast, no significant pooled effect was observed for the Short Physical Performance Battery (SPPB). While heterogeneity was moderate, meta-regression analyses failed to identify explanatory moderators. This finding should not be interpreted as evidence of intervention ineffectiveness or a limitation of the SPPB itself. Rather, most studies enrolled participants with high baseline SPPB scores, suggesting preserved functional capacity and a limited room for improvement, consistent with a ceiling effect.

For the Timed-Up and Go-Test (TUG), a significant moderate pooled effect (d = −0.51) reflected improvements in mobility and gait performance. The absence of substantial heterogeneity and publication bias suggests a relatively robust intervention effect, although meta-regression was again precluded by the limited number of studies.

Similarly, the chair rise test (CRT) showed a significant moderate effect (d = −0.34), accompanied by moderate heterogeneity. Meta-regression analyses did not identify significant moderators, and a substantial proportion of between-study variability remained unexplained.

Handgrip strength also demonstrated a significant moderate pooled effect (d = 0.33) with moderate heterogeneity, and no moderator effects were detected. Importantly, the consistent absence of significant moderator effects across outcomes should not be interpreted as evidence of comparable effectiveness across intervention modalities. Rather, this likely reflects limited statistical power and substantial within-category heterogeneity.

Exercise-based interventions encompassed a wide range of modalities, intensities, frequencies, durations, and progression schemes, which could not be systematically coded due to inconsistent reporting. This methodological limitation likely diluted intervention-specific effects and constrained the ability of meta-regression analyses to detect differential effectiveness.

Overall, the observed heterogeneity reflects both methodological diversity – related to intervention design, outcome selection, and measurement protocols – and clinical variability, including differences in diagnostic criteria, baseline functional status, and population characteristics. The lack of standardized intervention reporting and harmonized outcome selection fundamentally limits the clinical applicability of pooled estimates.

Consequently, although statistically significant effects were observed for several outcomes, their robustness and clinical interpretability remain constrained. Future studies should prioritize standardized reporting of intervention parameters, clearer definition of outcome measures, and harmonized outcome selection to facilitate more informative quantitative synthesis.

Beyond methodological considerations, an important conceptual question arises: from a patient perspective - are the most frequently assessed outcomes truly the most relevant? While body composition and strength are commonly measured, it remains unclear whether improvements in these surrogate endpoints translate into meaningful gains in functional independence, mobility, or quality of life. Evidence from patient preference studies suggest that mobility and the ability to manage daily activities are among the most valued outcomes [39], whereas expert perspectives often emphasize fall prevention [40]. The marked discrepancy between low inclusion of QoL measures (13 studies) and the frequent assessment of body composition (77 studies) highlights a persistent disconnect between trial outcomes and patient priorities. Addressing this gap requires a paradigm shift toward patient-centred outcome selection. Unlike surrogate measures, QoL instruments integrate functional, psychosocial and independence-related dimensions, providing a more comprehensive assessment of intervention benefits. This approach aligns with the GLIS 2025 consensus, which explicitly recommends prioritizing patient-relevant outcomes over surrogate measures [34]. Future trials should therefore systematically include sarcopenia-specific QoL instruments, such as SarQoL [37], and explicitly examine whether changes in muscle mass or strength correspond to meaningful improvements in patient-centered outcomes.

In this context, the development of Core Outcome Sets (COS) represents a crucial next step. The COMET initiative [41] provides a robust framework for the COS development through transparent, stakeholder-driven consensus processes [42]. Given the substantial heterogeneity observed across sarcopenia trials, COS implementation appears particularly urgent. Building on the findings of this review and experiences from related fields, COS development should integrate both commonly used outcome domains and patient-identified priorities, with broad stakeholder involvement. Feasibility considerations – including measurement burden, established psychometric properties, and clearly defined MCIDs – must be explicitly addressed to ensure implementability.

While updated ESCEO recommendations provide valuable guidance, they should be regarded as a initial step rather than a definitive COS, underscoring the need for a formal, patient-centred consensus process.

## 5 Limitations

This systematic review and meta-analysis has several limitations that should be acknowledged. A major challenge is the heterogeneity of the included interventions, which varied in type (e.g., nutritional strategies vs. physical training), duration, and specific implementation. In addition, considerable variability was observed in participant characteristics, including differences in diagnostic criteria for sarcopenia (e.g. EWGSOP, AWGS, FNIH), which apply distinct cut-off values and diagnostic constructs and may influence baseline status and responsiveness to interventions. Further heterogeneity arose from differences in age, living situations, and overall health status of the study population, thereby limiting the generalizability of the findings.

Comparability of the studies was further constrained by inconsistencies in outcome selection and measurement protocols, as different assessment methods were used for key variables. This variability may have affected the reliability of cross-study comparison and contributed to unexplained heterogeneity in pooled estimates. In addition, potential ceiling effects – particularly for the SPPB, the high baseline score reported in many studies – may have limited the sensitivity to detect intervention-related changes. For a methodological perspective, the limited numbers of studies available for several outcomes reduced the statistical power of moderator analyses and precluded meta-regression in some cases. Moreover, inconsistent and incomplete reporting of key intervention characteristics restricted the ability to systematically classify interventions and to explore differential effects across intervention types. Evidence of publication bias was identified for ASMI, suggesting that the corresponding pooled effect size may be overestimated.

Finally, the literature search strategy, including database selection and language restrictions, may have resulted in the omission of relevant studies. Variations in methodological quality and risk of bias across the included studies further limit the robustness of the conclusion.

Taken together, these limitations indicate that the present findings should be interpreted with caution. Future research would benefit from greater methodological harmonization, improved and standardized reporting of intervention protocols, consistent application of diagnostic criteria, and the development and implementation of core outcome sets to enhance comparability and strengthen the interpretability of evidence in sarcopenia research.

## 6 Conclusion

Our results underscore the pronounced heterogeneity of outcome measures in current sarcopenia trials, which substantially limits comparability across interventions and hinders evidence synthesis. These findings strongly support the need for a formally developed Core Outcome Set (COS) for sarcopenia that is aligned with patient priorities, incorporates clearly defined MCIDs, and balances scientific rigor with feasibility in clinical trials.

While the updated ESCEO recommendations represent an important step toward outcome standardization—particularly for phase III trials—they should be complemented and refined through a structured COS development process consistent with COMET methodology and inclusive stakeholder engagement. Establishing such a COS would provide a robust foundation for future sarcopenia research, enhance comparability across trials, and ultimately support high-quality meta-analyses as well as evidence-based clinical and regulatory decision-making.

## CRediT authorship contribution statement

M.R.: Conceptualization, Methodology, Formal analysis, Investigation, Writing – Original Draft, Visualization, Project administration, K.M.: Conceptualization, Methodology, Formal analysis, Investigation, Writing – Original Draft, Visualization, Project administration, S.M.: Writing – Review & Editing, R.S.: Writing – Review & Editing, O.T.: Writing – Review & Editing, S.S.: Writing – Review & Editing, L.D.: Writing – Review & Editing, M.D.: Conceptualization, Methodology, Resources, Writing – Review & Editing, Supervision.

## Ethical statement

As this study is a review, approval from institutional ethics committee was not required.

## Declaration of Generative AI and AI-assisted technologies in the writing process

During the preparation of this work the authors used OpenAI. (2025) (ChatGPT (GPT-5)) in order to improve the readability and language of the manuscript. After using this tool, the authors reviewed and edited the content as needed and take full responsibility for the content of the published article.

## Funding

For this project no funding was available.

## Data statement

Not applicable.

## Declaration of competing interest

All authors declare no conflict of interest.
