# Supplementary material for: Outcomes in clinical trials on sarcopenia: a systematic review and meta-analysis
Source: J Nutr Health Aging. 2026 Mar 3;30(4):100821. doi: 10.1016/j.jnha.2026.100821 (PMC12969113; doi:10.1016/j.jnha.2026.100821)
Supplement: Supplementary file 1 [file mmc1.docx]

| Table 1 List of included studies and main characteristics | | | | | | | | | | | |
| --- | --- | --- | --- | --- | --- | --- | --- | --- | --- | --- | --- |
| Authors | Pub year | sample size | country | Sarcopenia diagnosis | Age | Setting | Tool muscle measurement | Intervention type | Sarcopenia type | primary outcome(s) category | secondary outcome(s) category |
| Aleman-Mateo et al. (1) | 2012 | 40 | Mexico | other | ≥ 60 | community dwelling | DXA | nutrition | Sarcopenia | body composition physical performance | Other |
| Amasene et al (2) | 2022 | 41 | Spain | EWGSOP2 | ≥ 70 | post-hospitalized or geriatri outpatients | DXA | nutrition exercise / physical training | Sarcopenia | body composition | physical performance |
| Azevedo et al (3) | 2020 | 90 | Brazil | EWGSOP1 | not stated | other | other | nutrition exercise / physical training | Sarcopenia | other | other |
| Bagheri et al. (4) | 2020 | 30 | Iran | other | > 60 | other | BIA | exercise / physical training | Sarcopenia | other | body composition physical performance Other |
| Balachandran et al (5) | 2014 | 21 | USA | EWGSOP1 | 60-90 | community dwelling | BIA | exercise / physical training | Sarcopenic obesity | physical performance | physical performance body composition others |
| Bauer et al (6) | 2015 | 380 | 7 European countires | other | ≥ 65 | community dwelling | BIA | nutrition | Sarcopenia | physical performance | body composition QoL Other |
| Bellomo et al (7) | 2013 | 40 | Italy | other | not stated | other | other | exercise / physical training | Sarcopenia | physical performance |  |
| Bernabei et al (8) | 2022 | 1519 | 11 European countries | other | ≥ 70 | community dwelling | DXA | exercise / physical training | Sarcopnia probable Sarcopenia | physical performance | body composition physical performance |
| Björkman et al (9) | 2020 | 218 | finland | other | > 74 | community dwelling | other | nutrition | Sarcopenia | physical performance | body composition |
| Bo et al (10) | 2019 | 60 | china | other | 60-85 | not stated | BIA | nutrition | Sarcopenia | body composition physical performance other |  |
| Cebria et al (11) | 2018 | 81 | spain | other | >65 | nursing home | BIA | exercise | Sarcopenia | body composition physical performance |  |
| Chang et al (12) | 2021 | 57 | Taiwan | EWGSOP1 | >65 | community-dwelling | DXA | nutrition exercise / physical training | Sarcopenia | body composition physical performance | body composition physical performance |
| Chang et al (13) | 2018 | 17 | Taiwan | other | ≥ 60 | care homes | other | other | Sarcopenia | body composition physical performance QoL |  |
| Chen et al (14) | 2017 | 60 | Taiwan | other | 65-75 | sarcopenic obesity, willing to participate | BIA other | exercise / physical training | Sarcopenic obesity | body composition physical performance other |  |
| Chen et al (15) | 2018 | 33 | taiwan | AWGS1 | 65-75 | community dwelling | BIA | exercise / physical training | Sarcopenia | body composition physical performance other |  |
| Chiang et al (16) | 2021 | 35 | Taiwan | EWGSOP2 AWGS | > 75 | nursing home | BIA | nutrition exercise / physical training | Sarcopenia | body composition physical performance | Other |
| Chiu et al (17) | 2018 | 70 | Taiwan | other | ≥ 60 | long-term care | BIA | exercise / physical training | Sarcopenic obesity | body composition physical performance |  |
| Courel-Ibanez et al (18) | 2022 | 24 | Spain | FNIH | > 75 | care homes | DXA | exercise / physical training | Sarcopenia | physical performance | physical performance other |
| Cramer et al (19) | 2016 | 330 | 8 countries across Europe and North America | EWGSOP1 | ≥ 65 | community dwelling | DXA | nutrition | Sarcopenia | physical performance | body composition physical performance |
| da Cruz Alves et al (20) | 2022 | 34 | Brazil | EWGSOP1 | ≥ 65 | other[Follow-up: Outpatient Clinic] | BIA | nutrition exercise / physical training | Sarcopenia | body composition physical performance other |  |
| Damasceno et al (21) | 2019 | 53 | Brazil | EWGSOP1 | ≥ 60 | community dwelling | BIA | other | Sarcopenia probable Sarcopenia | body composition physical performance |  |
| El Hajj et al (22) | 2019 | 128 | lebanon | EWGSOP1 | not stated | hospitalized | BIA | nutrition | probable Sarcopenia | body composition physical performance other | body composition Other |
| Espinoza et al (23) | 2021 | 23 | USA (Texas) | EWGSOP1 | ≥ 60 | community dwelling | DXA | drug | Sarcopenia | body composition other | body composition physical performance Other |
| Ferhi et al (24) | 2023 | 40 | Tunisia | other | > 65 | other[Follow-up: obesity care centers] | BIA | exercise / physical training | Sarcopenic obesity | body composition physical performance other |  |
| Flor-Rufino et al (25) | 2023 | 51 | Spain | EWGSOP1 | ≥ 70 | community dwelling | BIA | exercise / physical training | Sarcopenia | body composition physical performance |  |
| Gadelha et al (26) | 2021 | 107 | Brazil | EWGSOP2 | ≥ 60 | other[Follow-up: hemodialysis] | DXA | exercise / physical training | Sarcopenia | other | body composition physical performance |
| Gadelha et al (27) | 2016 | 133 | Brazil | other | 60-80 | community dwelling | DXA | exercise / physical training | Sarcopenic obesity | body composition physical performance |  |
| Han et al (28) | 2022 | 200 | China | AWGS1 | ≥ 65 | hospitalized | DXA | nutrition exercise / physical training | Sarcopenia | body composition physical performance | Other |
| Hassan et al (29) | 2016 | 42 | Australia | EWGSOP1 | not stated | care homes | BIA | exercise / physical training | Sarcopenia | other | physical performance QoL Other |
| Huang et al (30) | 2017 | 35 | Taiwan | other | 60-90 | community dwelling | DXA BIA | exercise / physical training | Sarcopenic obesity | body composition other |  |
| Jung et al (31) | 2022 | 30 | Korea | AWGS1 | ≥ 65 | community dwelling | DXA | exercise / physical training | Sarcopenic obesity | body composition other |  |
| Jung et al (32) | 2019 | 26 | Korea | AWGS1 | ≥ 65 | community dwelling | DXA BIA | exercise / physical training | Sarcopenia | body composition physical performance other |  |
| Kemmler et al (33) | 2016 | 75 | Germany | EWGSOP1 | ≥ 70 | community dwelling | DXA | other | Sarcopenic obesity | body composition | body composition physical performance |
| Kemmler et al (34) | 2020 | 100 | Germany | FNIH | ≥ 70 | community dwelling | other | nutrition exercise / physical training | Sarcopenia | other |  |
| Kemmler et al (35) | 2017 | 100 | Germany | EWGSOP1 | ≥ 70 | community dwelling | BIA | nutrition other | Sarcopenic obesity | body composition | body composition physical performance |
| Kim et al (36) | 2016 | 139 | Japan | other | ≥ 70 | community dwelling | BIA | nutrition exercise / physical training | Sarcopenic obesity | body composition physical performance other |  |
| Kim et al (37) | 2013 | 128 | Japan | other | ≥ 75 | community dwelling | BIA | nutrition exercise / physical training | Sarcopenia | body composition physical performance |  |
| Kim et al (38) | 2012 | 155 | Japan | other | ≥ 75 | community dwelling | BIA | nutrition exercise / physical training | Sarcopenia | body composition physical performance |  |
| Lee et al (39) | 2021 | 27 | Taiwan | EWGSOP1 | 60-90 | community dwelling | DXA | exercise / physical training | Sarcopenic obesity | body composition physical performance other |  |
| Li et al (40) | 2020 | 241 | China | AWGS1 | ≥ 60 | community dwelling | BIA | nutrition exercise / physical training | Sarcopenia | body composition physical performance |  |
| Li et al (41) | 2022 | 70 | China | AWGS1 | 60-80 | care homes/hospital | not stated | exercise / physical training | Sarcopenia | other | physical performance |
| Liang et al (42) | 2020 | 60 | China | AWGS1 | >80 | acute care unit at hospital | BIA | exercise / physical training | Sarcopenia | physical performance other | physical performance Other |
| Liao et al (43) | 2017 | 46 | Taiwan | EWGSOP1 | 60-80 | other[Follow-up: outpatient department of rehabilitation center at the hospital] | DXA BIA | exercise / physical training | Sarcopenic obesity | body composition physical performance other |  |
| Lichtenberg et al (44) | 2019 | 43 | Germany | other | ≥ 72 | community dwelling | DXA | nutrition exercise / physical training | Sarcopenia Sarcopenic obesity | body composition | body composition physical performance |
| Lin et al (45) | 2021 | 56 | Taiwan | AWGS1 | ≥ 65 | other[Follow-up: tertiary medical center hospital] | BIA | nutrition | Sarcopenia | body composition physical performance |  |
| Liu et al (46) | 2014 | 177 | USA | other | 70-89 | no info | DXA | exercise / physical training other | Sarcopenia | physical performance |  |
| Lu et al (47) | 2021 | 246 | Singapore | AWGS1 | ≥ 65 | community dwelling | DXA | nutrition exercise / physical training | Sarcopenia | body composition physical performance |  |
| Lu et al (48) | 2019 | 92 | Singapore | AWGS1 | ≥ 65 | community dwelling | DXA | nutrition exercise / physical training other | Sarcopenia | body composition physical performance other |  |
| Makizako et al (49) | 2020 | 72 | japan | AWGS1 | ≥ 60 | community dwelling | MRI | exercise / physical training | Sarcopenia probable Sarcopenia | body composition physical performance |  |
| Maltais et al (50) | 2016 | 26 | Canada | other | 60-75 | community dwelling | DXA | nutrition | Sarcopenia | body composition physical performance |  |
| Martinez-Arnau et al (51) | 2020 | 50 | Spain | EWGSOP1 | ≥ 65 | care homes | BIA | nutrition | Sarcopenia | body composition physical performance other | Other |
| Meza-Valderrama et al (52) | 2024 | 32 | Spain | EWGSOP2 | ≥ 60 | other | BIA | drug exercise / physical training | Sarcopenia | physical performance | body composition QoL Other |
| Monti et al (53) | 2023 | 45 | 11 European countries | FNIH | ≥ 70 | community dwelling | DXA | exercise / physical training | Sarcopenia | physical performance |  |
| Morawin et al (54) | 2021 | 80 | Poland | EWGSOP2 | 60-90 | community dwelling other | BIA | exercise / physical training | Sarcopenia | body composition physical performance other |  |
| Mori H, Tokuda Y (55) | 2022 | 81 | Japan | AWGS1 | ≥ 65 | other | BIA | nutrition exercise / physical training | Sarcopenia | body composition physical performance |  |
| Murphy et al (56) | 2022 | 83 | Ireland | EWGSOP1 | ≥ 65 | community dwelling | DXA | nutrition | Sarcopenia probable Sarcopenia | body composition | body composition physical performance Other |
| Nabuco et al (57) | 2019 | 26 | Brazil | FNIH | ≥ 60 | community dwelling | DXA | nutrition exercise / physical | Sarcopenic obesity | body composition physical performance other |  |
| Nasimi et al (58) | 2021 | 66 | Iran | AWGS1 | ≥ 65 | community dwelling | DXA | nutrition | Sarcopenia | body composition physical performance other | physical performance QoL Other |
| Nilsson et al (59) | 2020 | 45 | Canada | EWGSOP1 | ≥ 65 | community dwelling | DXA | nutrition exercise / physical training | Sarcopenia | body composition physical performance other |  |
| Oh et al (60) | 2020 | 38 | Korea | AWGS1 | 65-90 | hospitalized | DXA | exercise / physical training | Sarcopenia | physical performance | physical performance QoL other |
| Osuka et al (61) | 2021 | 156 | Japan | AWGS1 | ≥ 65 | other[Follow-up: population-based screening] | BIA | nutrition exercise / physical training | Sarcopenia | body composition | physical performance Other |
| Papanicolaou et al (62) | 2013 | 170 | Brazil, Chile, Colombia, Denmark, Finland, France, Hong Kong, Israel, Mexico, New Zealand, Peru, Soth Africa, Spain, Sweden, UK | other | ≥ 65 |  | DXA | drug | Sarcopenia probable Sarcopenia | body composition physical performance other |  |
| Piastra et al (63) | 2018 | 72 | italy | EWGSOP1 | ≥ 65 | community dwelling | BIA | exercise / physical training | Sarcopenia | body composition physical performance other |  |
| Rondanelli et al (64) | 2019 | 159 | Italy | EWGSOP1 | ≥ 65 | other | DXA | nutrition exercise / physical training | Sarcopenia | body composition physical performance other |  |
| Rondanelli et al (65) | 2020 | 140 | Italy | EWGSOP1 | ≥ 65 | other | DXA BIA | nutrition exercise / physical training | Sarcopenia | physical performance | physical performance |
| Rondanelli et al (66) | 2016 | 130 | Italy | other | ≥ 65 | other | DXA | nutrition exercise / physical training | Sarcopenia | body composition | body composition physical performance QoL Other |
| Rooks et al (67) | 2020 | 180 | North America, France, Germany, Denmark, Belgium, Spain, Switzerland, Japan, Taiwan, Czech Republic, Russia, South Korea | EWGSOP1 AWGS | ≥ 70 | community dwelling | DXA | drug nutrition exercise / physical training | Sarcopenia | physical performance | body composition physical performance |
| Seo et al (68) | 2021 | 22 | South Korea | EWGSOP1 | ≥ 65 | community dwelling other | DXA | exercise / physical training | Sarcopenia | body composition physical performance other | physical performance QoL |
| Silva et al (69) | 2018 | 49 | Brasil | EWGSOP1 | ≥ 60 | community dwelling | DXA | exercise / physical training | Sarcopenic obesity | body composition physical performance |  |
| Takeuchi et al (70) | 2019 | 68 | Japan | AWGS1 | ≥ 65 | other | other | nutrition exercise / physical training | Sarcopenia | physical performance | body composition physical performance Other |
| Tamura et al (71) | 2023 | 83 | Japan | EWGSOP1 AWGS | ≥ 65 |  | BIA | nutrition | Sarcopenia | body composition | body composition physical performance QoL Other |
| Tokuda Y, Mori H (72) | 2023 | 54 | Japan | AWGS2 | ≥ 65 | community dwelling | BIA | nutrition exercise / physical training | Sarcopenia | body composition | physical performance QoL |
| Tsekoura et al (73) | 2018 | 54 | Greece | EWGSOP1 | ≥ 60 | community dwelling | BIA | exercise / physical training | Sarcopenia probable Sarcopenia | body composition physical performance QoL |  |
| Tung et al (74) | 2022 | 103 | Taiwan | AWGS2 | ≥ 65 | care homes |  | exercise / physical training | probable sarcopenia | physical performance other |  |
| Vasconcelos et al (75) | 2016 | 28 | Brazil | other | 65-80 |  |  | exercise / physical training | Sarcopenic obesity | physical performance QoL |  |
| Vijayakumaran R, Daly R, Tan V (76) | 2023 | 16 | Malaysia | AWGS2 | ~ 66 | community dwelling | DXA | nutrition exercise / physical training | Sarcopenia probable sarcopenia | body composition physical performance other |  |
| Vikberg et al (77) | 2019 | 70 | Sweden | EWGSOP1 | 70 | community dwelling | DXA | exercise / physical training | probable Sarcopenia | physical performance | body composition physical performance |
| Wang et al (78) | 2022 | 201 | China | EWGSOP2/ AWGS | 65-75 | other | BIA | nutrition exercise / physical training | Sarcopenia | body composition physical performance |  |
| Wei et al (79) | 2022 | 90 | China | AWGS1 | 60-75 | community dwelling other | CT | exercise / physical training | Sarcopenia | body composition physical performance |  |
| Wei et al (80) | 2017 | 80 | Hong Kong | other | ≥ 65 | community dwelling | BIA | exercise / physical training | Sarcopenia | physical performance |  |
| Witham et al (81) | 2022 | 145 | UK | EWGSOP1 | ≥ 70 | community dwelling hospitalized other | BIA | drug nutrition | Sarcopenia | physical performance | body composition QoL Other |
| Yamada et al (82) | 2019 | 112 | Japan | AWGS1 | ≥ 65 | community dwelling | BIA | nutrition exercise / physical training | Sarcopenia | body composition physical performance |  |
| Yang et al (83) | 2023 | 34 | China | AWGS2 | ≥ 60 | community dwelling | BIA | nutrition exercise / physical training | Sarcopenia | physical performance | body composition physical performance Other |
| Yin et al (84) | 2023 | 60 | China | AWGS2 | ≥ 60 | community dwelling | BIA | nutrition | Sarcopenic obesity | body composition physical performance other |  |
| Yoshimura et al (85) | 2019 | 44 | Japan | AWGS1 | ≥ 65 | hospitalized other | BIA | nutrition exercise / physical training | Sarcopenia | physical performance | body composition physical performance |
| Yuenyongchaiwat K, Akekawatchai C (86) | 2022 | 57 | Thailand | AWGS2 | ≥ 60 | community dwelling | BIA | exercise / physical training | Sarcopenia | body composition physical performance other |  |
| Zdzieblik et al (87) | 2015 | 53 | Germany | EWGSOP1 | > 65 | community dwelling | DXA | nutrition exercise / physical training | Sarcopenia | body composition | body composition physical performance |
| Zhou et al (88) | 2018 | 48 | China | AWGS1 | > 60 | community dwelling | BIA | nutrition other | Sarcopenic obesity | body composition |  |
| Zhu et al (89) | 2019 | 113 | China | AWGS1 | ≥ 65 | community dwelling other | DXA | nutrition exercise / physical training | Sarcopenia | physical performance | body composition physical performance QoL Other |
| Zhu et al (90) | 2019 | 79 | China | AWGS1 | ≥ 85 | other | DXA | exercise / physical training | Sarcopenic obesity | body composition physical performance |  |

*Table 2: Detailed Search Strategy*

| MEDLINE via Pubmed | elder* OR aged OR geriat* AND sarcopen* AND Randomized Controlled Trial AND English, German |
| --- | --- |
| EMBASE via Ovid | elder* OR aged OR geriat* AND sarcopen* AND randomized controlled trials  Limits: human  Language: English, German |
| Web of Science | elder* OR aged OR geriat* AND sarcopen* AND randomized controlled trial AND English OR German |
| Psychinfo | elder* OR aged OR geriat* And sarcopen*  Limits: Language: English, German; Methodology: Clinical trial |
| Clinical trial registers (ICTRP, Clinical Trials.gov, CINAHL) via Cochrane Library | elder* OR aged OR geriat* AND sarcopen* AND controlled trial OR rct OR controlled clinical trial OR randomized trial OR randomized controlled trial OR groups OR trial OR double blind OR random*  Limits: English, German |

Table 3 MCID

| Measurement | Studies using MCID |
| --- | --- |
| Gait speed | Balachandran et al. (2014), Meza-Valderrama et al. (2024), Zhu_1 et al. (2019) |
| SPPB | Courel-Ibanez et al. (2022), Meza-Valderrama et al. (2024), Rooks et al. (2020), Witham et al. (2022) |
| Composite strength | Murphy et al. (2022) |
| TUG | Murphy et al. (2022) |
| Quadriceps strength | Witham et al. (2022) |
| Handgrip strength | Witham et al. (2022) |
| 6-min. walking distance | Witham et al. (2022) |
| Barthel Index | Liang et al. (2020) |
| Fat Free Mass | Gadelha et al. (2016), Rondanelli et al. (2016) |
| Sarcopenia Z-Score | Lichtenberg et al. (2019) |
| Appendicular Lean Mass | Murphy et al. (2022) |
| Lean Mass | Nasimi et al. (2021) |
| Skeletal Muscle Index | Tamura et al. (2023) |
| Physical performance | Balachandran et al. (2014), Meza-Valderrama et al. (2024), Zhu_1 et al. (2019), Courel-Ibanez et al. (2022), Rooks et al. (2020), Witham et al. (2022), Liang et al. (2020), Murphy et al. (2022) |
| Body composition | Gadelha et al. (2016), Rondanelli et al. (2016), Lichtenberg et al. (2019), Murphy et al. (2022), Nasimi et al. (2021), Tamura et al. (2023) |

eFigure 1

eFigure 1a shows the applied sarcopenia definitions and the number of studies based on these definitions

eFigure 1b shows the different types of sarcopenia studies and the number of studies in which each definition was applied

eFigure 2

eFigure 2 shows the number of studies using different physical performance and muscle strength measurements

eFigure 3

eFigure 3 shows the number of studies using different body composition measurements

References

1. Alemán-Mateo H, Macías L, Esparza-Romero J, Astiazaran-García H, Blancas AL. Physiological effects beyond the significant gain in muscle mass in sarcopenic elderly men: evidence from a randomized clinical trial using a protein-rich food. Clin Interv Aging. 2012;7:225-34.

2. Amasene M, Cadenas-Sanchez C, Echeverria I, Sanz B, Alonso C, Tobalina I, et al. Effects of Resistance Training Intervention along with Leucine-Enriched Whey Protein Supplementation on Sarcopenia and Frailty in Post-Hospitalized Older Adults: Preliminary Findings of a Randomized Controlled Trial. J Clin Med. 2021;11(1).

3. Pinheiro HA, Cerceau VR, Pereira LC, Funghetto SS, Menezes RLd. Nutritional intervention and functional exercises improve depression, loneliness and quality of life in elderly women with sarcopenia: a randomized clinical trial. Fisioterapia em Movimento. 2020;33.

4. Bagheri R, Moghadam BH, Church DD, Tinsley GM, Eskandari M, Moghadam BH, et al. The effects of concurrent training order on body composition and serum concentrations of follistatin, myostatin and GDF11 in sarcopenic elderly men. Exp Gerontol. 2020;133:110869.

5. Balachandran A, Krawczyk SN, Potiaumpai M, Signorile JF. High-speed circuit training vs hypertrophy training to improve physical function in sarcopenic obese adults: a randomized controlled trial. Exp Gerontol. 2014;60:64-71.

6. Bauer JM, Verlaan S, Bautmans I, Brandt K, Donini LM, Maggio M, et al. Effects of a vitamin D and leucine-enriched whey protein nutritional supplement on measures of sarcopenia in older adults, the PROVIDE study: a randomized, double-blind, placebo-controlled trial. J Am Med Dir Assoc. 2015;16(9):740-7.

7. Bellomo RG, Iodice P, Maffulli N, Maghradze T, Coco V, Saggini R. Muscle Strength and Balance Training in Sarcopenic Elderly: A Pilot Study with Randomized Controlled Trial. European Journal of Inflammation. 2013;11(1):193-201.

8. Bernabei R, Landi F, Calvani R, Cesari M, Del Signore S, Anker SD, et al. Multicomponent intervention to prevent mobility disability in frail older adults: randomised controlled trial (SPRINTT project). Bmj. 2022;377:e068788.

9. Björkman MP, Suominen MH, Kautiainen H, Jyväkorpi SK, Finne-Soveri HU, Strandberg TE, et al. Effect of Protein Supplementation on Physical Performance in Older People With Sarcopenia-A Randomized Controlled Trial. J Am Med Dir Assoc. 2020;21(2):226-32.e1.

10. Bo Y, Liu C, Ji Z, Yang R, An Q, Zhang X, et al. A high whey protein, vitamin D and E supplement preserves muscle mass, strength, and quality of life in sarcopenic older adults: A double-blind randomized controlled trial. Clin Nutr. 2019;38(1):159-64.

11. Cebrià IIM, Balasch-Bernat M, Tortosa-Chuliá M, Balasch-Parisi S. Effects of Resistance Training of Peripheral Muscles Versus Respiratory Muscles in Older Adults With Sarcopenia Who are Institutionalized: A Randomized Controlled Trial. J Aging Phys Act. 2018;26(4):637-46.

12. Chang KV, Wu WT, Huang KC, Han DS. Effectiveness of early versus delayed exercise and nutritional intervention on segmental body composition of sarcopenic elders - A randomized controlled trial. Clin Nutr. 2021;40(3):1052-9.

13. Chang SF, Lin PC, Yang RS, Yang RJ. The preliminary effect of whole-body vibration intervention on improving the skeletal muscle mass index, physical fitness, and quality of life among older people with sarcopenia. BMC Geriatr. 2018;18(1):17.

14. Chen HT, Chung YC, Chen YJ, Ho SY, Wu HJ. Effects of Different Types of Exercise on Body Composition, Muscle Strength, and IGF-1 in the Elderly with Sarcopenic Obesity. J Am Geriatr Soc. 2017;65(4):827-32.

15. Chen HT, Wu HJ, Chen YJ, Ho SY, Chung YC. Effects of 8-week kettlebell training on body composition, muscle strength, pulmonary function, and chronic low-grade inflammation in elderly women with sarcopenia. Exp Gerontol. 2018;112:112-8.

16. Chiang FY, Chen JR, Lee WJ, Yang SC. Effects of Milk or Soy Milk Combined with Mild Resistance Exercise on the Muscle Mass and Muscle Strength in Very Old Nursing Home Residents with Sarcopenia. Foods. 2021;10(11).

17. Chiu SC, Yang RS, Yang RJ, Chang SF. Effects of resistance training on body composition and functional capacity among sarcopenic obese residents in long-term care facilities: a preliminary study. BMC Geriatr. 2018;18(1):21.

18. Courel-Ibáñez J, Buendía-Romero Á, Pallarés JG, García-Conesa S, Martínez-Cava A, Izquierdo M. Impact of Tailored Multicomponent Exercise for Preventing Weakness and Falls on Nursing Home Residents' Functional Capacity. J Am Med Dir Assoc. 2022;23(1):98-104.e3.

19. Cramer JT, Cruz-Jentoft AJ, Landi F, Hickson M, Zamboni M, Pereira SL, et al. Impacts of High-Protein Oral Nutritional Supplements Among Malnourished Men and Women with Sarcopenia: A Multicenter, Randomized, Double-Blinded, Controlled Trial. J Am Med Dir Assoc. 2016;17(11):1044-55.

20. da Cruz Alves NM, Pfrimer K, Santos PC, de Freitas EC, Neves T, Pessini RA, et al. Randomised Controlled Trial of Fish Oil Supplementation on Responsiveness to Resistance Exercise Training in Sarcopenic Older Women. Nutrients. 2022;14(14).

21. Soares Mendes Damasceno G, Teixeira T, de Souza VC, Neiva TS, Prudente Pereira K, Teles Landim MF, et al. Acupuncture Treatment in Elderly People with Sarcopenia: Effects on the Strength and Inflammatory Mediators. J Aging Res. 2019;2019:8483576.

22. El Hajj C, Fares S, Chardigny JM, Boirie Y, Walrand S. Vitamin D supplementation and muscle strength in pre-sarcopenic elderly Lebanese people: a randomized controlled trial. Arch Osteoporos. 2018;14(1):4.

23. Espinoza SE, Lee JL, Wang CP, Ganapathy V, MacCarthy D, Pascucci C, et al. Intranasal Oxytocin Improves Lean Muscle Mass and Lowers LDL Cholesterol in Older Adults with Sarcopenic Obesity: A Pilot Randomized Controlled Trial. J Am Med Dir Assoc. 2021;22(9):1877-82.e2.

24. Ferhi H, Gaied Chortane S, Durand S, Beaune B, Boyas S, Maktouf W. Effects of Physical Activity Program on Body Composition, Physical Performance, and Neuromuscular Strategies during Walking in Older Adults with Sarcopenic Obesity: Randomized Controlled Trial. Healthcare (Basel). 2023;11(16).

25. Flor-Rufino C, Barrachina-Igual J, Pérez-Ros P, Pablos-Monzó A, Sanz-Requena R, Martínez-Arnau FM. Fat infiltration and muscle hydration improve after high-intensity resistance training in women with sarcopenia. A randomized clinical trial. Maturitas. 2023;168:29-36.

26. Gadelha AB, Cesari M, Corrêa HL, Neves RVP, Sousa CV, Deus LA, et al. Effects of pre-dialysis resistance training on sarcopenia, inflammatory profile, and anemia biomarkers in older community-dwelling patients with chronic kidney disease: a randomized controlled trial. Int Urol Nephrol. 2021;53(10):2137-47.

27. Gadelha AB, Paiva FM, Gauche R, de Oliveira RJ, Lima RM. Effects of resistance training on sarcopenic obesity index in older women: A randomized controlled trial. Arch Gerontol Geriatr. 2016;65:168-73.

28. Han Z, Ji NN, Ma JX, Dong Q, Ma XL. Effect of Resistance Training Combined with Beta-Hydroxy-Beta-Methylbutyric Acid Supplements in Elderly Patients with Sarcopenia after Hip Replacement. Orthop Surg. 2022;14(4):704-13.

29. Hassan BH, Hewitt J, Keogh JW, Bermeo S, Duque G, Henwood TR. Impact of resistance training on sarcopenia in nursing care facilities: A pilot study. Geriatr Nurs. 2016;37(2):116-21.

30. Huang SW, Ku JW, Lin LF, Liao CD, Chou LC, Liou TH. Body composition influenced by progressive elastic band resistance exercise of sarcopenic obesity elderly women: a pilot randomized controlled trial. Eur J Phys Rehabil Med. 2017;53(4):556-63.

31. Jung WS, Kim YY, Kim JW, Park HY. Effects of Circuit Training Program on Cardiovascular Risk Factors, Vascular Inflammatory Markers, and Insulin-like Growth Factor-1 in Elderly Obese Women with Sarcopenia. Rev Cardiovasc Med. 2022;23(4):134.

32. Jung WS, Kim YY, Park HY. Circuit Training Improvements in Korean Women with Sarcopenia. Percept Mot Skills. 2019;126(5):828-42.

33. Kemmler W, Teschler M, Weissenfels A, Bebenek M, von Stengel S, Kohl M, et al. Whole-body electromyostimulation to fight sarcopenic obesity in community-dwelling older women at risk. Resultsof the randomized controlled FORMOsA-sarcopenic obesity study. Osteoporos Int. 2016;27(11):3261-70.

34. Kemmler W, von Stengel S, Kohl M, Rohleder N, Bertsch T, Sieber CC, et al. Safety of a Combined WB-EMS and High-Protein Diet Intervention in Sarcopenic Obese Elderly Men. Clin Interv Aging. 2020;15:953-67.

35. Kemmler W, Weissenfels A, Teschler M, Willert S, Bebenek M, Shojaa M, et al. Whole-body electromyostimulation and protein supplementation favorably affect sarcopenic obesity in community-dwelling older men at risk: the randomized controlled FranSO study. Clin Interv Aging. 2017;12:1503-13.

36. Kim H, Kim M, Kojima N, Fujino K, Hosoi E, Kobayashi H, et al. Exercise and Nutritional Supplementation on Community-Dwelling Elderly Japanese Women With Sarcopenic Obesity: A Randomized Controlled Trial. J Am Med Dir Assoc. 2016;17(11):1011-9.

37. Kim H, Suzuki T, Saito K, Yoshida H, Kojima N, Kim M, et al. Effects of exercise and tea catechins on muscle mass, strength and walking ability in community-dwelling elderly Japanese sarcopenic women: a randomized controlled trial. Geriatr Gerontol Int. 2013;13(2):458-65.

38. Kim HK, Suzuki T, Saito K, Yoshida H, Kobayashi H, Kato H, et al. Effects of exercise and amino acid supplementation on body composition and physical function in community-dwelling elderly Japanese sarcopenic women: a randomized controlled trial. J Am Geriatr Soc. 2012;60(1):16-23.

39. Lee YH, Lee PH, Lin LF, Liao CD, Liou TH, Huang SW. Effects of progressive elastic band resistance exercise for aged osteosarcopenic adiposity women. Exp Gerontol. 2021;147:111272.

40. Li Z, Cui M, Yu K, Zhang XW, Li CW, Nie XD, et al. Effects of nutrition supplementation and physical exercise on muscle mass, muscle strength and fat mass among sarcopenic elderly: a randomized controlled trial. Appl Physiol Nutr Metab. 2021;46(5):494-500.

41. Li ZR, Ma YJ, Zhuang J, Tao XC, Guo CY, Liu ST, et al. Ditangquan exercises based on safe-landing strategies prevent falls and injury among older individuals with sarcopenia. Front Med (Lausanne). 2022;9:936314.

42. Liang Y, Wang R, Jiang J, Tan L, Yang M. A randomized controlled trial of resistance and balance exercise for sarcopenic patients aged 80-99 years. Sci Rep. 2020;10(1):18756.

43. Liao CD, Tsauo JY, Lin LF, Huang SW, Ku JW, Chou LC, et al. Effects of elastic resistance exercise on body composition and physical capacity in older women with sarcopenic obesity: A CONSORT-compliant prospective randomized controlled trial. Medicine (Baltimore). 2017;96(23):e7115.

44. Lichtenberg T, von Stengel S, Sieber C, Kemmler W. The Favorable Effects of a High-Intensity Resistance Training on Sarcopenia in Older Community-Dwelling Men with Osteosarcopenia: The Randomized Controlled FrOST Study. Clin Interv Aging. 2019;14:2173-86.

45. Lin CC, Shih MH, Chen CD, Yeh SL. Effects of adequate dietary protein with whey protein, leucine, and vitamin D supplementation on sarcopenia in older adults: An open-label, parallel-group study. Clin Nutr. 2021;40(3):1323-9.

46. Liu CK, Leng X, Hsu FC, Kritchevsky SB, Ding J, Earnest CP, et al. The impact of sarcopenia on a physical activity intervention: the Lifestyle Interventions and Independence for Elders Pilot Study (LIFE-P). J Nutr Health Aging. 2014;18(1):59-64.

47. Lu Y, Niti M, Yap KB, Tan CTY, Nyunt MSZ, Feng L, et al. Effects of multi-domain lifestyle interventions on sarcopenia measures and blood biomarkers: secondary analysis of a randomized controlled trial of community-dwelling pre-frail and frail older adults. Aging (Albany NY). 2021;13(7):9330-47.

48. Lu Y, Niti M, Yap KB, Tan CTY, Zin Nyunt MS, Feng L, et al. Assessment of Sarcopenia Among Community-Dwelling At-Risk Frail Adults Aged 65 Years and Older Who Received Multidomain Lifestyle Interventions: A Secondary Analysis of a Randomized Clinical Trial. JAMA Netw Open. 2019;2(10):e1913346.

49. Makizako H, Nakai Y, Tomioka K, Taniguchi Y, Sato N, Wada A, et al. Effects of a Multicomponent Exercise Program in Physical Function and Muscle Mass in Sarcopenic/Pre-Sarcopenic Adults. J Clin Med. 2020;9(5).

50. Maltais ML, Ladouceur JP, Dionne IJ. The Effect of Resistance Training and Different Sources of Postexercise Protein Supplementation on Muscle Mass and Physical Capacity in Sarcopenic Elderly Men. J Strength Cond Res. 2016;30(6):1680-7.

51. Martínez-Arnau FM, Fonfría-Vivas R, Buigues C, Castillo Y, Molina P, Hoogland AJ, et al. Effects of Leucine Administration in Sarcopenia: A Randomized and Placebo-controlled Clinical Trial. Nutrients. 2020;12(4).

52. Meza-Valderrama D, Sánchez-Rodríguez D, Messaggi-Sartor M, Muñoz-Redondo E, Morgado-Pérez A, Tejero-Sánchez M, et al. Supplementation with β-hydroxy-β-methylbutyrate after resistance training in post-acute care patients with sarcopenia: A randomized, double-blind placebo-controlled trial. Arch Gerontol Geriatr. 2024;119:105323.

53. Monti E, Tagliaferri S, Zampieri S, Sarto F, Sirago G, Franchi MV, et al. Effects of a 2-year exercise training on neuromuscular system health in older individuals with low muscle function. J Cachexia Sarcopenia Muscle. 2023;14(2):794-804.

54. Morawin B, Tylutka A, Chmielowiec J, Zembron-Lacny A. Circulating Mediators of Apoptosis and Inflammation in Aging; Physical Exercise Intervention. Int J Environ Res Public Health. 2021;18(6).

55. Mori H, Tokuda Y. De-Training Effects Following Leucine-Enriched Whey Protein Supplementation and Resistance Training in Older Adults with Sarcopenia: A Randomized Controlled Trial with 24 Weeks of Follow-Up. J Nutr Health Aging. 2022;26(11):994-1002.

56. Murphy CH, Connolly C, Flanagan EM, Mitchelson KAJ, de Marco Castro E, Egan B, et al. Interindividual variability in response to protein and fish oil supplementation in older adults: a randomized controlled trial. J Cachexia Sarcopenia Muscle. 2022;13(2):872-83.

57. Nabuco HCG, Tomeleri CM, Fernandes RR, Sugihara Junior P, Cavalcante EF, Cunha PM, et al. Effect of whey protein supplementation combined with resistance training on body composition, muscular strength, functional capacity, and plasma-metabolism biomarkers in older women with sarcopenic obesity: A randomized, double-blind, placebo-controlled trial. Clin Nutr ESPEN. 2019;32:88-95.

58. Nasimi N, Sohrabi Z, Dabbaghmanesh MH, Eskandari MH, Bedeltavana A, Famouri M, et al. A Novel Fortified Dairy Product and Sarcopenia Measures in Sarcopenic Older Adults: A Double-Blind Randomized Controlled Trial. J Am Med Dir Assoc. 2021;22(4):809-15.

59. Nilsson MI, Mikhail A, Lan L, Di Carlo A, Hamilton B, Barnard K, et al. A Five-Ingredient Nutritional Supplement and Home-Based Resistance Exercise Improve Lean Mass and Strength in Free-Living Elderly. Nutrients. 2020;12(8).

60. Oh MK, Yoo JI, Byun H, Chun SW, Lim SK, Jang YJ, et al. Efficacy of Combined Antigravity Treadmill and Conventional Rehabilitation After Hip Fracture in Patients With Sarcopenia. J Gerontol A Biol Sci Med Sci. 2020;75(10):e173-e81.

61. Osuka Y, Kojima N, Sasai H, Wakaba K, Miyauchi D, Tanaka K, et al. Effects of exercise and/or β-hydroxy-β-methylbutyrate supplementation on muscle mass, muscle strength, and physical performance in older women with low muscle mass: a randomized, double-blind, placebo-controlled trial. Am J Clin Nutr. 2021;114(4):1371-85.

62. Papanicolaou DA, Ather SN, Zhu H, Zhou Y, Lutkiewicz J, Scott BB, et al. A phase IIA randomized, placebo-controlled clinical trial to study the efficacy and safety of the selective androgen receptor modulator (SARM), MK-0773 in female participants with sarcopenia. J Nutr Health Aging. 2013;17(6):533-43.

63. Piastra G, Perasso L, Lucarini S, Monacelli F, Bisio A, Ferrando V, et al. Effects of Two Types of 9-Month Adapted Physical Activity Program on Muscle Mass, Muscle Strength, and Balance in Moderate Sarcopenic Older Women. Biomed Res Int. 2018;2018:5095673.

64. Rondanelli M, Peroni G, Gasparri C, Infantino V, Nichetti M, Cuzzoni G, et al. Is a Combination of Melatonin and Amino Acids Useful to Sarcopenic Elderly Patients? A Randomized Trial. Geriatrics (Basel). 2018;4(1).

65. Rondanelli M, Cereda E, Klersy C, Faliva MA, Peroni G, Nichetti M, et al. Improving rehabilitation in sarcopenia: a randomized-controlled trial utilizing a muscle-targeted food for special medical purposes. J Cachexia Sarcopenia Muscle. 2020;11(6):1535-47.

66. Rondanelli M, Klersy C, Terracol G, Talluri J, Maugeri R, Guido D, et al. Whey protein, amino acids, and vitamin D supplementation with physical activity increases fat-free mass and strength, functionality, and quality of life and decreases inflammation in sarcopenic elderly. Am J Clin Nutr. 2016;103(3):830-40.

67. Rooks D, Swan T, Goswami B, Filosa LA, Bunte O, Panchaud N, et al. Bimagrumab vs Optimized Standard of Care for Treatment of Sarcopenia in Community-Dwelling Older Adults: A Randomized Clinical Trial. JAMA Netw Open. 2020;3(10):e2020836.

68. Seo MW, Jung SW, Kim SW, Lee JM, Jung HC, Song JK. Effects of 16 Weeks of Resistance Training on Muscle Quality and Muscle Growth Factors in Older Adult Women with Sarcopenia: A Randomized Controlled Trial. Int J Environ Res Public Health. 2021;18(13).

69. de Oliveira Silva A, Dutra MT, de Moraes W, Funghetto SS, Lopes de Farias D, Dos Santos PHF, et al. Resistance training-induced gains in muscle strength, body composition, and functional capacity are attenuated in elderly women with sarcopenic obesity. Clin Interv Aging. 2018;13:411-7.

70. Takeuchi I, Yoshimura Y, Shimazu S, Jeong S, Yamaga M, Koga H. Effects of branched-chain amino acids and vitamin D supplementation on physical function, muscle mass and strength, and nutritional status in sarcopenic older adults undergoing hospital-based rehabilitation: A multicenter randomized controlled trial. Geriatr Gerontol Int. 2019;19(1):12-7.

71. Tamura Y, Kaga H, Abe Y, Yoshii H, Seino H, Hiyoshi T, et al. Efficacy and Safety of 5-Aminolevulinic Acid Combined with Iron on Skeletal Muscle Mass Index and Physical Performance of Patients with Sarcopenia: A Multicenter, Double-Blinded, Randomized-Controlled Trial (ALADDIN Study). Nutrients. 2023;15(13).

72. Tokuda Y, Mori H. Essential Amino Acid and Tea Catechin Supplementation after Resistance Exercise Improves Skeletal Muscle Mass in Older Adults with Sarcopenia: An Open-Label, Pilot, Randomized Controlled Trial. J Am Nutr Assoc. 2023;42(3):255-62.

73. Tsekoura M, Billis E, Tsepis E, Dimitriadis Z, Matzaroglou C, Tyllianakis M, et al. The Effects of Group and Home-Based Exercise Programs in Elderly with Sarcopenia: A Randomized Controlled Trial. J Clin Med. 2018;7(12).

74. Tung HT, Chen KM, Huang KC, Hsu HF, Chou CP, Kuo CF. Effects of Vitality Acupunch exercise on functional fitness and activities of daily living among probable sarcopenic older adults in residential facilities. J Nurs Scholarsh. 2022;54(2):176-83.

75. Vasconcelos KS, Dias JM, Araújo MC, Pinheiro AC, Moreira BS, Dias RC. Effects of a progressive resistance exercise program with high-speed component on the physical function of older women with sarcopenic obesity: a randomized controlled trial. Braz J Phys Ther. 2016;20(5):432-40.

76. Vijayakumaran RK, Daly RM, Tan VPS. "We want more": perspectives of sarcopenic older women on the feasibility of high-intensity progressive resistance exercises and a whey-protein nutrition intervention. Front Nutr. 2023;10:1176523.

77. Vikberg S, Sörlén N, Brandén L, Johansson J, Nordström A, Hult A, et al. Effects of Resistance Training on Functional Strength and Muscle Mass in 70-Year-Old Individuals With Pre-sarcopenia: A Randomized Controlled Trial. J Am Med Dir Assoc. 2019;20(1):28-34.

78. Wang Z, Xu X, Gao S, Wu C, Song Q, Shi Z, et al. Effects of Internet-Based Nutrition and Exercise Interventions on the Prevention and Treatment of Sarcopenia in the Elderly. Nutrients. 2022;14(12).

79. Wei M, Meng D, Guo H, He S, Tian Z, Wang Z, et al. Hybrid Exercise Program for Sarcopenia in Older Adults: The Effectiveness of Explainable Artificial Intelligence-Based Clinical Assistance in Assessing Skeletal Muscle Area. Int J Environ Res Public Health. 2022;19(16).

80. Wei N, Pang MY, Ng SS, Ng GY. Optimal frequency/time combination of whole body vibration training for developing physical performance of people with sarcopenia: a randomized controlled trial. Clin Rehabil. 2017;31(10):1313-21.

81. Witham MD, Adamson S, Avenell A, Band MM, Bashir T, Donnan PT, et al. Efficacy and Mechanism Evaluation. Leucine and perindopril to improve physical performance in people over 70 years with sarcopenia: the LACE factorial RCT. Southampton (UK): National Institute for Health and Care Research

Copyright © 2022 Witham et al. This work was produced by Witham et al. under the terms of a commissioning contract issued by the Secretary of State for Health and Social Care. This is an Open Access publication distributed under the terms of the Creative Commons Attribution CC BY 4.0 licence, which permits unrestricted use, distribution, reproduction and adaption in any medium and for any purpose provided that it is properly attributed. See: <https://creativecommons.org/licenses/by/4.0/>. For attribution the title, original author(s), the publication source – NIHR Journals Library, and the DOI of the publication must be cited.; 2022.

82. Yamada M, Kimura Y, Ishiyama D, Nishio N, Otobe Y, Tanaka T, et al. Synergistic effect of bodyweight resistance exercise and protein supplementation on skeletal muscle in sarcopenic or dynapenic older adults. Geriatr Gerontol Int. 2019;19(5):429-37.

83. Yang C, Song Y, Li T, Chen X, Zhou J, Pan Q, et al. Effects of Beta-Hydroxy-Beta-Methylbutyrate Supplementation on Older Adults with Sarcopenia: A Randomized, Double-Blind, Placebo-Controlled Study. J Nutr Health Aging. 2023;27(5):329-39.

84. Yin YH, Liu JYW, Välimäki M. Dietary behaviour change intervention for managing sarcopenic obesity among community-dwelling older people: a pilot randomised controlled trial. BMC Geriatr. 2023;23(1):597.

85. Yoshimura Y, Bise T, Shimazu S, Tanoue M, Tomioka Y, Araki M, et al. Effects of a leucine-enriched amino acid supplement on muscle mass, muscle strength, and physical function in post-stroke patients with sarcopenia: A randomized controlled trial. Nutrition. 2019;58:1-6.

86. Yuenyongchaiwat K, Akekawatchai C. Beneficial effects of walking-based home program for improving cardio-respiratory performance and physical activity in sarcopenic older people: a randomized controlled trial. Eur J Phys Rehabil Med. 2022;58(6):838-44.

87. Zdzieblik D, Oesser S, Baumstark MW, Gollhofer A, König D. Collagen peptide supplementation in combination with resistance training improves body composition and increases muscle strength in elderly sarcopenic men: a randomised controlled trial. Br J Nutr. 2015;114(8):1237-45.

88. Zhou X, Xing B, He G, Lyu X, Zeng Y. The Effects of Electrical Acupuncture and Essential Amino Acid Supplementation on Sarcopenic Obesity in Male Older Adults: A Randomized Control Study. Obes Facts. 2018;11(4):327-34.

89. Zhu LY, Chan R, Kwok T, Cheng KC, Ha A, Woo J. Effects of exercise and nutrition supplementation in community-dwelling older Chinese people with sarcopenia: a randomized controlled trial. Age Ageing. 2019;48(2):220-8.

90. Zhu YQ, Peng N, Zhou M, Liu PP, Qi XL, Wang N, et al. Tai Chi and whole-body vibrating therapy in sarcopenic men in advanced old age: a clinical randomized controlled trial. Eur J Ageing. 2019;16(3):273-82.
